# When automation hits jobs: Entrepreneurship as an alternative career path

**DOI:** 10.1371/journal.pone.0331244

**Published:** 2025-09-08

**Authors:** Daehyun Kim, Taekyun Kim, Wonjoon Kim, Hyejin Youn

**Affiliations:** 1 Max Planck Institute for Innovation and Competition, München, Germany; 2 School of Business, Chungnam National University, Daejeon, South Korea; 3 School of Business and Technology Management, KAIST, Daejeon, South Korea; 4 College of Business Administration, Seoul National University, Seoul, South Korea; Universidade Federal do Tocantins, BRAZIL

## Abstract

This study investigates the relationship between occupational automation risks and workers’ transitions to entrepreneurship using data from the Current Population Survey. We find that employees facing automation-related job displacement are inclined to shift toward unincorporated entrepreneurship, emphasizing entrepreneurship as a viable alternative career path. Noteworthy variations emerge when examining specific automation technologies, revealing a positive association between industrial robots and entrepreneurial transitions, whereas artificial intelligence displays a negative relationship. Gender disparities are observed, with female workers exhibiting a lower likelihood than males of transitioning into entrepreneurship. This study also shows a heightened prominence of entrepreneurial transitions during the early stages of the COVID-19 pandemic. By illuminating entrepreneurship as a response to job displacement, our results offer crucial policy insights into the labor market implications of automation.

## 1 Introduction

The increasing adoption of robotics and artificial intelligence (AI) in business processes generates significant gains in productivity and economic potential [1], but this benefit is not without its trade-off; it simultaneously generates opportunities for growth and risks of job displacement [2,3]. As automation redefines tasks traditionally performed solely by humans to be executed by machines [4], crowding out human labors reignites discussions about technological unemployment due to the march of machines [5]. This issue is especially pertinent for automation-susceptible occupations that have been empirically shown to be more vulnerable to job displacement [6].

The pace at which automation is reshaping the labor landscape is unprecedented, and the full spectrum of its implications on workforce adaptation remains to be fully elucidated [7]. Notably, roles that require high levels of skill, once thought to be insulated from the risks of automation, are now seeing a shift toward entrepreneurship when automation endangers their jobs [8]. This indicates that entrepreneurship may serve as an underexplored outlet in the automation-job displacement narrative. Accordingly, in this paper we investigate how the risk of job automation is correlated with transitions into entrepreneurship, distinguishing between opportunity- and necessity-driven motives. We further examine how this relationship differs across demographic factors, such as gender, and environmental contexts, such as the COVID-19 pandemic.

In this study, we propose that employees facing a high risk of being displaced by automation technology are more inclined to consider entrepreneurship. However, we argue that such entrepreneurship is not driven by opportunity but by necessity, arising from being pushed out of traditional paid employment and used as a last resort in the face of unemployment. As a result, it is more likely to result in unincorporated self-employment, often chosen by unemployed individuals with lower levels of education [9] and typically characterized by its smaller scale and limited scope for innovation [10]. We further assert that the likelihood of transitioning to necessity entrepreneurship is more pronounced among employees who are extensively exposed to automation technologies that mechanize routine tasks rather than technologies that augment human abilities. We also explore gender dynamics within this context, suggesting that female workers, often perceived as more risk-averse, may be more reluctant to engage in necessity entrepreneurship, which tends to have a higher failure rate on average [9]. Finally, set against the backdrop of the COVID-19 pandemic, a period marked by accelerated adoption of automation for workplace safety and business continuity, we anticipate an increase in transitions to necessity entrepreneurship as a response to the disruptive reduction of employment opportunities.

By matching individuals across consecutive monthly CPS datasets, we can observe changes in their employment status, specifically identifying which legal form of self-employment they have transitioned into. Following prior research [11–13], we classify incorporated self-employment as opportunity entrepreneurship and unincorporated self-employment as necessity entrepreneurship. This more detailed classification, reflecting recent discussions in the literature on the heterogeneity of self-employment, is essential for examining how exposure to automation technologies correlates with the type of entrepreneurial transition. [14] In addition, we used the occupational automation risk measure developed by Frey and Osborne [4] to assess the vulnerability of occupations to automation. As automation technologies include diverse forms, including industrial robots and AI, we also integrated occupational exposure to these technologies as established by Webb [15] and Felten et al. [16]. This approach aims to uncover worker’s tendencies in the move toward entrepreneurship in relation to exposure to different automation technologies.

From the analysis, we found that employees with occupations at risk of being displaced by automation are likely to transition to necessity entrepreneurship, whereas no significant correlation was found with opportunity entrepreneurship. Our analysis further revealed that the increased likelihood of unemployment for workers in automation-prone occupations might drive this trend, highlighting the role of entrepreneurship as an alternative career option. We also found a diverse association between occupation’s exposure to automation technologies and entrepreneurial transition. Specifically, our results indicate that distinct automation technologies exhibit varied relationships with entrepreneurial transitions: industrial robots are positively associated with necessity entrepreneurship, while AI exposure is negatively associated with necessity entrepreneurship. Additionally, we observed that female workers are less likely to transition into entrepreneurship. Finally, our analysis revealed that the prominence of entrepreneurial transition was particularly evident in the early stage of the COVID-19 pandemic.

This paper contributes to the existing literature in many ways. First, it builds upon and expands the discussions initiated by Fossen and Sorgner [8] by exploring the relationship between workers’ responses to automation risks and their transition into entrepreneurship. Specifically, we show that workers exposed to different types of automation technology may choose varied career trajectories. Second, our research provides important insights into how individuals’ risk attitudes toward entrepreneurship can moderate the relationship between workers’ exposure to automation and their entrepreneurial choices, with a particular focus on demographic factors like gender. Finally, we show that environmental changes can be a moderating factor for those susceptible to automation in choosing entrepreneurship as a career path.

## 2 Theoretical background

We first discuss the relationship between occupational risks to automation technology and employees’ decisions to engage in entrepreneurship. Then we explore the heterogeneity in the relationship, considering factors such as different types of automation technologies (e.g., industrial robotics, AI), gender, and environmental changes such as the COVID-19 pandemic.

### 2.1 Automation technology and entrepreneurship

Considerable improvements in automation technologies such as industrial robots and AI have affected workers in many occupations by performing tasks traditionally considered feasible only by human labor. In line with this, recent studies have investigated the relationship between automation and employment [17,18]. Although some authors have argued that automation can create new jobs [19], there has been much rhetoric regarding job destruction narratives. For example, approximately half of all occupations have the potential for automation, leading to a transformation in the employment landscape through the integration of automation technologies [4,20,21]. In this vein, automation can influence workers’ occupation choices [8]. In particular, as workers in automation-prone occupations face a high probability of displacement, the continuous adoption of automation technologies can be related with these individuals’ entrepreneurship choices.

When individuals consider becoming entrepreneurs, the availability of employment opportunities in the labor market plays a crucial role in shaping that decision [22]. Prior research in the entrepreneurship literature has demonstrated that transitions into self-employment often occur when conventional employment options become scarce [23,24]. In particular, when opportunities for salaried work decline, individuals may turn to entrepreneurship as an alternative pathway for sustaining income. Thus, the state of the labor market can influence entrepreneurial entry decisions [25]. For example, studies have shown that people are more likely to pursue entrepreneurship in contexts of labor market discrimination [26] or during periods of rising unemployment [27].

We contend that entrepreneurship entry can be an alternative for employees who are at risk of displacement by automation technology. The changes induced by automation technology, particularly in the areas of automating manual and routine tasks traditionally performed by human labor, have reshaped the fabric of workplaces [28]. Specifically, given that the adoption of automation technologies can frequently result in mass layoffs [29], it can pose challenges for the existing labor market in absorbing displaced workers. Similarly, Fujita [30] underscores the observable decline in employment opportunities within the labor market for roles involving manual and routine tasks. This paradigm shift sets the stage for an environment where workers, predominantly endowed with manual and routine skills, confront both a heightened risk of displacement [31] and difficulties in securing alternative employment through conventional channels.

In essence, entrepreneurship often emerges as a potential pathway for workers confronting automation risks. However, not all forms of entrepreneurship reflect the same motivations or outcomes. Prior research highlights a crucial distinction between opportunity entrepreneurship and necessity entrepreneurship [11–14]. Opportunity entrepreneurship occurs when individuals voluntarily pursue new ventures to fulfill higher-order goals, such as self-actualization, autonomy, or the desire to exploit new market possibilities [11,12]. These ventures are typically growth-oriented, innovative, and are often structured as incorporated businesses with formalized operations and the potential for scalability. This form of entrepreneurship aligns with traditional narratives of entrepreneurs as proactive agents seeking to create new wealth and capture market opportunities. In contrast, necessity entrepreneurship arises when individuals are pushed into self-employment to meet basic needs, such as maintaining income after job loss or coping with limited access to traditional employment [12,13]. These ventures frequently unincorporated, small in scale, and oriented toward subsistence rather than innovation or expansion. As noted by [11], necessity entrepreneurs often create businesses that replicate existing services or products, rather than introducing novel offerings, and typically operate with limited growth potential. This form of entrepreneurship serves as a survival strategy rather than a pathway to wealth creation.

Following this distinction, we predict that individuals facing the risk of displacement due to automation are more likely to transition into necessity entrepreneurship. These workers may turn to entrepreneurship not because of market opportunities but due to limited alternatives in the labor market. In other words, workers at risk of losing their jobs to automation are often pushed toward entrepreneurship not as a process of starting new ventures exploiting market opportunities, but rather as an alternative path forced by the diminishing opportunities for salaried work in the labor market. Consequently, rather than establishing innovative businesses that offer new technologies and products to consumers in the market, those at risk from automation are more likely to transition into unincorporated businesses, which are typically smaller and less innovative [10]. Accordingly, we predict that workers vulnerable to automation are more likely to transition into necessity entrepreneurship as a forced response to displacement.

### 2.2 Different types of automation technologies and entrepreneurship

Our discussion emphasizes the risk of worker displacement due to automation, potentially driving employees toward entrepreneurship. It is crucial to acknowledge that distinct aspects of automation technologies, like industrial robots and AI, influence entrepreneurial choices based on the nuanced nature of tasks, distinguishing between routine and cognitive functions [32].

The integration of industrial robots in workplaces mechanizes routine tasks traditionally performed by human labor, enhancing efficiency but potentially leading to job displacement [17]. With the advancement of robotics technology, there’s a foreseeable consequence of not only displacing human workers but also limiting job opportunities in the labor market [30]. Faced with redundancy and a shrinking job market, individuals may turn to entrepreneurship for economic survival. Meanwhile, AI operates differently, augmenting human capabilities in cognitive tasks and decision-making rather than entirely displacing them [33]. Roles involving collaboration with AI can lead to an enhancement of job functions, creating a synergy between human and AI skills [34]. This collaborative dynamic reduces the likelihood of job loss, thereby diminishing the inclination of individuals toward entrepreneurial endeavors because their roles remain complementary rather than competitive with AI technologies. Furthermore, the current state of AI is not advanced enough for complete human displacement [35], emphasizing the collaborative and complementary nature of human-AI interactions in the workplace.

In summary, exposure to industrial robots and AI is likely to guide workers along different entrepreneurial pathways. Employees exposed to industrial robots are more inclined to pursue entrepreneurship as an alternative choice, whereas those exposed to AI technologies are less likely to embark on entrepreneurship.

### 2.3 Automation technology and entrepreneurship across gender

In Section 2.3, we discuss how the relationship between workers’ exposure to automation risk and their inclination toward entrepreneurship varies depending on gender. We predict that female employees who face the threat of job displacement by automation technologies are less likely to transition into unincorporated business than their male counterparts. The literature suggests that women tend to exhibit a higher degree of risk aversion than their male counterparts [36]. This characteristic, particularly relevant in the context of entrepreneurship’s inherent high failure rates, might deter women from engaging in entrepreneurial activities to the same extent as men. This gender-based disparity in attitudes toward entrepreneurship becomes even more pronounced in the context of unincorporated entrepreneurship.

Considering that females tend to exhibit a higher risk-averse tendency than males, and given the increased failure rates of unincorporated businesses initiated due to unemployment [9], it is plausible that female workers vulnerable to automation are less likely to transition into unincorporated business as an alternative career path than their male counterparts. This gender-based difference in risk aversion could play a significant role in influencing their decision against pursuing entrepreneurship in the form of unincorporated businesses, particularly in situations where job security is threatened by automation. Therefore, we argue that female workers whose jobs are at risk of automation are less likely to transition to unincorporated business than men in similar automation-prone situations.

### 2.4 Automation technology and entrepreneurship during COVID-19

**Fig 1:**
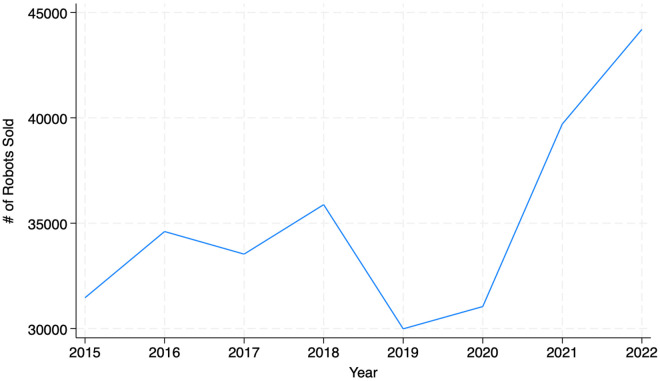
The demand for industrial robots in North American (2015–2022). Source: Association for Advancing Automation (2023).

Section 2.4 explores the relationship between workers’ exposure to automation risk and their inclination toward entrepreneurship during the COVID-19 pandemic. We argue that automation-prone workers’ transition to entrepreneurship can be prominent during the pandemic because employers have rapidly adopted automation technology to protect the workplace environment. The implementation of social-distancing measures during the pandemic necessitated changes in business operations, sparking a widespread shift toward automation [37]. According to a survey conducted by McKinsey [38], two-thirds of 800 senior executives reported investing in automation to lessen physical proximity and manage rising demand. The trend was further supported by a marked increase in the demand for robots in North America, growing by 4%, 28%, and 11% in 2020, 2021 and 2022, respectively, as shown in Fig 1.

Despite being a strategic response to navigate the crisis, this accelerated adoption of technology during the pandemic has raised concerns about potential job displacement and transformations in labor market conditions [39]. For example, a survey on individuals’ views on automation during the pandemic showed that employees believe their jobs are at risk of being lost in the near future [40]. In practice, reductions in new hiring were pronounced among jobs that involved routine tasks such as handicraft and printing [41] and sales [42]. In this distressed labor market, employees vulnerable to automation might face increasing difficulties in finding salaried job opportunities. Consequently, as COVID-19-induced automation eliminated viable job opportunities, particularly in roles requiring routine and manual tasks, these displaced workers became more inclined to pursue entrepreneurial activities as an alternative career path.

## 3 Data, variables, and estimation method

### 3.1 Data

In our study, we used data from the CPS covering January 2015 to September 2023 to track individual entrepreneurial activities. This survey, which sampled U.S. households, gathered detailed information on individuals, including education, labor status, and demographics. The CPS follows a rotating survey design, where households are interviewed for four months, followed by an eight-month break, and then interviewed again for another four months. Table S1 in our study details this rotating pattern to clarify the structure and dynamics of the CPS data.

The CPS’s survey design allows researchers to match consecutive observations of individuals, effectively creating a rotating panel dataset. For example, according to S1 Table, when comparing January of year *t* with February of year *t* (or any two adjacent months), we observe that 75% of the CPS sample remains consistent from one month to the next. Using this rotation pattern, we link the CPS monthly files over time with household and individual identifiers. To ensure the accuracy of the matched CPS files, we rigorously checked for any incorrect matches by comparing details like race, sex, and age in consecutive months’ data. In doing so, we created a dataset that allowed us to understand individual month-to-month labor mobility.

These matched CPS files contain a total of 9,564,290 observations, among which there were 1,999,643 unique individuals. Because our research primarily focused on workers threatened by job displacement due to automation technology, we limited our sample to workers aged 18–64 employed in private companies. This resulted in approximately 2,296,191 observations, of which 731,078 are of unique individuals who worked at private companies. Using this sample, we tracked whether private workers transition into entrepreneurs in the subsequent survey month and measured the extent to which these workers were exposed to the threat of job displacement due to automation technology. Details of this approach will be discussed in Section 3.2.1. Main Dependent Variables.

### 3.2 Variable description

#### 3.2.1 Main dependent variables.

Consistent with prior work (e.g., [27]), we defined entrepreneurship as individuals who make a transition to self-employment. The measure is widely used to identify an individual’s entrepreneurial activities using CPS data [8,27]. To measure individual transitions to entrepreneurship in the matched CPS database, we identified all individuals 18–64 years old who worked at private companies in the first survey month in the two-month pair. Then we identified whether these workers made transitions from wage work to self-employment as their primary job in the following month.

From these processes, we captured workers’ detailed entrepreneurial activities by distinguishing between opportunity and necessity entrepreneurship, based on the legal form of self-employment. Thus, we code two separate dependent variables: necessity entrepreneurship is coded as one if employees transition to unincorporated self-employment in the following month, and opportunity entrepreneurship is coded as one if they transition to incorporated self-employment; both are coded as zero otherwise.

Consistent with the literature, we considered incorporated businesses that those who are self-employed form as a proxy for formal and high-scalable entrepreneurship, contrasting to unincorporated businesses [10,27]. Guzman and Stern [43] tested this assumption, finding that incorporated businesses are more than three times as likely to achieve higher growth, resulting in an initial public offering or acquisition. Levine and Rubinstein [44] also provided evidence that those who form an incorporated business engage in activities that require a high degree of inventiveness, analytical flexibility, and general problem-solving. In comparison, unincorporated firms created under the same conditions are more likely to engage in activities that require a relatively low level of cognitive skills and to be small in size [10]. Moreover, low-educated unemployed individuals are more likely to engage in self-employment through unincorporated businesses, which typically have a lower innovation potential [9]. Accordingly, we classified incorporated self-employment as opportunity entrepreneurship, which tends to be more formal and growth-oriented, and unincorporated self-employment as necessity entrepreneurship, which is typically informal and less growth-oriented.

Alongside tracking individuals’ entrepreneurial activities, our research focused on whether employees at private companies transition to unemployment. Using a similar approach to that employed for identifying transitions into entrepreneurship, we tracked whether workers shifted from wage employment to unemployment in the following month. This measure explored whether workers in automation-prone occupations were more susceptible to job loss.

#### 3.2.2 Main independent variables.

Our main independent variable of interest was the susceptibility of workers to automation technology, which we measured using the automation probability index (API) established by Frey and Osborne [4]. They developed automation probabilities to quantify the risk of human job displacement by machines. This measurement is designed to capture the risk of job displacement by estimating the probability of computerization in 702 detailed occupations over the next 10–20 years by relying on expert opinion and features of selected occupations in the O*NET database (see more detail in the S2 Table).

To determine if the API measure accurately represents the risk of occupations being replaced by automation technology, we compared it with the findings of previous studies. To do this, we examined the relationship between the API and occupational routineness using four occupational routine measures developed by Acemoglu and Autor [45]. These measures are composite indices derived from the O*NET Work Activities and Work Context Importance scales. More detailed information on these measures, which represent the sum of their respective constituent scales standardized to a mean of zero and a standard deviation of one, can be found in the S3 Table [45].

**Fig 2:**
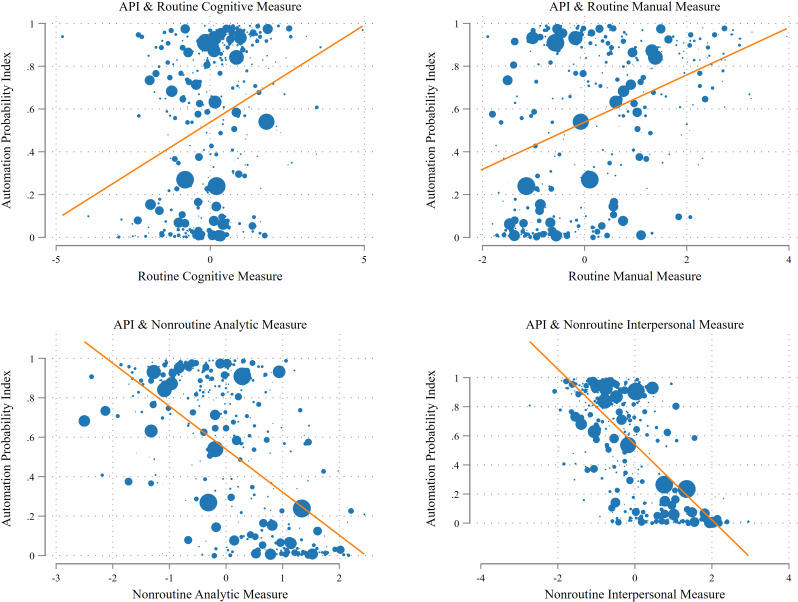
Automation probability index (API) and O*NET task measures. This figure displays scatterplots summarizing relationships between the automation probability index and O*NET task measures. The size of each marker increases as the number of overlapping data points increases. The automation probability index is sourced from Frey and Osborne [4], and O*NET task measures are sourced from Acemoglu and Autor [45].

From the analysis, we found that the measure of API provides a meaningful gauge of the automation vulnerability of different occupational groups. Fig 2 demonstrates a clear relationship: occupations with a higher frequency of routine cognitive and routine manual tasks tend to have higher API scores. In contrast, those with more nonroutine analytic and nonroutine interpersonal tasks usually show lower API scores. This pattern aligns with Autor et al. [46], who observed a correlation between computerization and a reduction in labor input for routine tasks alongside an increase for nonroutine analytics and nonroutine interactive tasks.

Furthermore, we examined occupational exposure to industrial robots and AI, two prominent automation technologies known for their significant impacts on the economy and society, among various types of automation. First, to quantify exposure to industrial robots, we used Webb’s method [15]. This method calculates the exposure for each occupation by identifying overlaps between industrial robot patents and job tasks outlined in the O*NET (see more detail in the S2 Table). The author used verb–noun pairs from patent text data and O*NET to quantify this overlap. As a result, occupations with a higher proportion of overlapping tasks were considered more exposure to industrial robots. We used this measure to determine each occupation’s robot exposure score.

Second, we measured workers’ exposure to AI with two established measurements. The first was from Webb’s measure [15], which also captures occupations’ AI exposure measure (see more detail in the S2 Table). The mechanism of calculating how occupation exposure to AI technology is the same we used to explain exposure to industrial robots. We used Felten et al.’s measurement [16] established from the AI Progress Measurement project by the Electronic Frontier Foundation. These data identify 10 AI progress areas since 2010, mapped to the 52 O*NET ability scales. Then the AI occupational impact is calculated for each O*NET occupation as the weighted sum of 52 AI application-ability scores. Thus, we used these measurements to calculate the AI exposure score for each occupation.

We applied these measurements (automation probability index, robot exposure score, and AI exposure score) to our matched CPS sample, using the six-digit system of occupational classification (SOC) codes. We were able to match the automation probability index from Frey and Osborne to 1,409,990 observations, Webb’s measures to 1,512,965 observations, and the Felten et al. measures to 1,512,965 observations. Our main sample, focused on the relationship between workers’ susceptibility to automation and their entrepreneurial activities, was constructed using the matched CPS data and the measurements from Frey and Osborne, comprising 1,409,990 observations.

#### 3.2.3 Control variables.

We used several control variables in our empirical analysis. First, we controlled for workers’ education level, which is related to their recognition of a business opportunity [47]. We also included a set of variables to control for the respondent’s age, age squared, sex, marital status, education, citizenship, and household income, which might affect the decision to start a business [48,49]. In addition, we included fixed effects for state-year, industry, and quarter to control for seasonality and the state-level monthly unemployment rate to better reflect the socioeconomic environment. Basic descriptive statistics for our main sample are presented in Table 1.

**Table 1:** Summary statistics for main analyses.

	Obs.	Mean	SD	Min	Max
Unemployment	1,409,990	0.03503	0.18386	0	1
Necessity Entrepreneurship	1,409,990	0.00301	0.05477	0	1
Opportunity Entrepreneurship	1,409,990	0.00129	0.03584	0	1
API (Frey and Osborne)	1,409,990	0.53057	0.36606	0.0028	.99
Robot exposure (Webb)	1,512,965	0.47839	0.58789	0.00795	3.09446
AI exposure (Webb)	1,512,965	0.38813	0.22742	0	1.48831
AI exposure (Felten et al.)	1,512,965	0.10632	1.01238	–2.11195	1.52606
Age	1,409,990	40.34053	12.84753	18	64
Sex	1,409,990	1.45658	0.49811	1	2
Ethnicity	1,409,990	2.13439	1.89561	1	6
Marital Status	1,409,990	0.5291	0.49915	0	1
Education	1,409,990	3.38968	1.50886	1	6
Citizenship	1,409,990	0.83781	0.36862	0	1
Household Income	1,409,990	12.49672	3.35108	1	16
State Unemp. rate	1,409,990	4.8402	1.9697	1.9	29.5
Industry (NAICS 3 Digit)	1,409,990	490.6618	160.94849	111	814
State	1,409,990	25.21332	15.14714	1	51
Year	1,409,990	2018.4586	2.4	2015	2023
Quarter (Seasonality)	1,409,990	2.44321	1.12185	1	4

Descriptions for each of the variables. Sex: 1 for male, 2 for female. Ethnicity: 1 for White, 2 for Black, 3 for Native American, 4 for Asian, 5 for multiracial, 6 for Hispanic. Marital status: 0 for not married, 1 for married. Education: 1 for less than high school, 2 for high school, 3 for some college, 4 for associate degree, 5 for bachelor’s degree, 6 for graduate degree. Citizenship: 0 for not a U.S. citizen, 1 for U.S. citizen. Household income: household income category in CPS. Industry: industries are classified as first three digits of a firm’s North American Industry Classification System (NAICS) code.

### 3.3 Estimation method

For the main analyses, we used the linear probability model for the separate dichotomous dependent variables. Our main empirical design was as follows:


$$Y_{ikst}=\varphi_{st}+\sigma_{k}+\lambda_{t}+{\beta_{1}X}_{it}+{\beta_{2}\gamma }_{i}+{\beta_{3}C}_{st}+{\beta_{4}API}_{i}+\varepsilon_{ist},$$

where $Y_{ikst}$ is the main dependent variable that represents the labor mobility of individuals *i* in an industry *k* in a state *s* in year *t*;$\;\phi_{st}$ is a state–year fixed effect; $\sigma_{k}$ is an industry fixed effect; $\lambda_{t}$ represents a quarter-fixed effect to control for seasonality; $X_{it}$ is a vector of time-variant individual-level controls (age, age squared, household income, marital status, and education); $\gamma_{i}$ is a vector of time-invariant individual-level controls (ethnicity, citizenship, sex); $C_{st}$ refers to state-level monthly unemployment rate; and ${API}_{i}$ are our main independent variables. Standard errors are clustered at the occupation level to address any heteroskedasticity and serial correlation concerns. Finally, we used individual-level sampling weights obtained from CPS in our estimates.

## 4 Results

### 4.1 Relationship between API and unemployment/entrepreneurship

**Fig 3:**
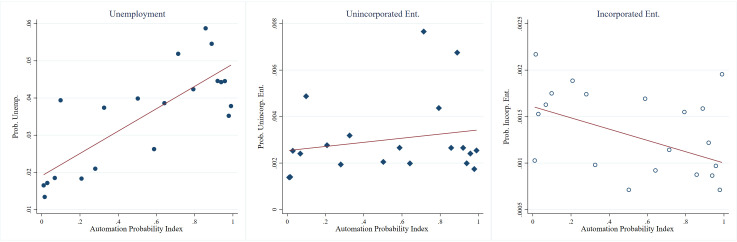
Unconditional relationship between automation probability index (API) and Labor Mobility. This figure displays binned scatterplots summarizing the relationships between API and labor mobility, which include transitions to unemployment, unincorporated self-employment, and incorporated self-employment. The automation probability index is sourced from Frey and Osborne [4].

First, we examined the unconditional relationship between the API and labor mobility. Fig 3 illustrates the association between API and workers’ transitions into unemployment and different forms of entrepreneurship. First, the figure shows a positive correlation between the API and the probability of workers’ transition into unemployment. This trend indicates that occupations with a higher API are more likely to see their workers transition into unemployment. Similarly, there is a positive correlation between API and the likelihood of workers’ becoming unincorporated self-employed. Meanwhile, we observed a negative correlation between API and the transition of workers into incorporated self-employment. In conclusion, these findings indicate a clear pattern: workers in high-API occupations tend to move toward unemployment and unincorporated self-employment while showing less inclination toward incorporated self-employment.

Table 2 presents the results from our linear probability model that examines the association between the API and the transition of workers into unemployment or entrepreneurship. This analysis is pivotal in understanding how varying degrees of automation risk, as quantified by the API, correlate with shifts in employment status. As indicated in the first column of Table 2, the analysis reveals that there is a positive correlation between API and the probability of unemployment. Specifically, a one-standard-deviation increase in API is linked with a 1.262 percentage point rise in the likelihood of unemployment. In relative terms, given that the baseline unemployment rate is about 3.503%, this corresponds to a 36.026% increase in the unemployment rate. Taken together, we found that employees whose occupations are more likely to be automated have a higher likelihood of becoming unemployed in subsequent survey months.

**Table 2:** Automation Probability Index (API), Unemployment, and Entrepreneurship.

DV	Unemployment	Necessity Ent.	Opportunity Ent.
	(1)	(2)	(3)
API	0.01262^***^	0.00078^**^	–0.00012
	(0.00219)	(0.00034)	(0.00019)
Controls:			
Individual	Yes	Yes	Yes
State unemp. rate	Yes	Yes	Yes
Fixed effects:			
Industry	Yes	Yes	Yes
State # year	Yes	Yes	Yes
Seasonality	Yes	Yes	Yes
N	1,409,990	1,409,990	1,409,990
Adj. R^2^	0.02346	0.00355	0.00083

Sample is employees who work for private companies. Robust standard errors, adjusted for clustering at the occupation level, are presented in parentheses. Individual-level control variables are family income, citizenship, age, age squared, sex, ethnicity, marital status, education, citizenship, and household income. The automation probability index is sourced from Frey and Osborne. * p < 0.1, ** p < 0.05, *** p < 0.01

We further explored the relationship between the API and the transition to both necessity and opportunity entrepreneurship. As indicated in the second column of Table 2, there exists a positive correlation between the API and the likelihood of transitioning into necessity entrepreneurship. More specifically, our findings suggest that a one-standard-deviation increase in the API corresponds to approximately a 0.078% increase in the probability of workers’ transitioning into necessity entrepreneurship. This increase represents 25.914% of the monthly baseline probability of entry into this form of entrepreneurship, which stands at 0.301%. However, our analysis did not reveal a statistically significant relationship between the API and the transition into opportunity entrepreneurship. These results imply that workers in occupations vulnerable to automation are more likely to transition into unincorporated rather than incorporated self-employment.

In summary, our analyses led us to observe that workers in occupations with a high API are more likely to transition into unemployment and to engage in unincorporated self-employment. This supports our theoretical prediction that posits that workers at risk of job displacement due to automation technology are more likely to consider necessity entrepreneurship as an alternative choice.

### 4.2 Relationship between automation technologies and unemployment/entrepreneurship

**Fig 4:**
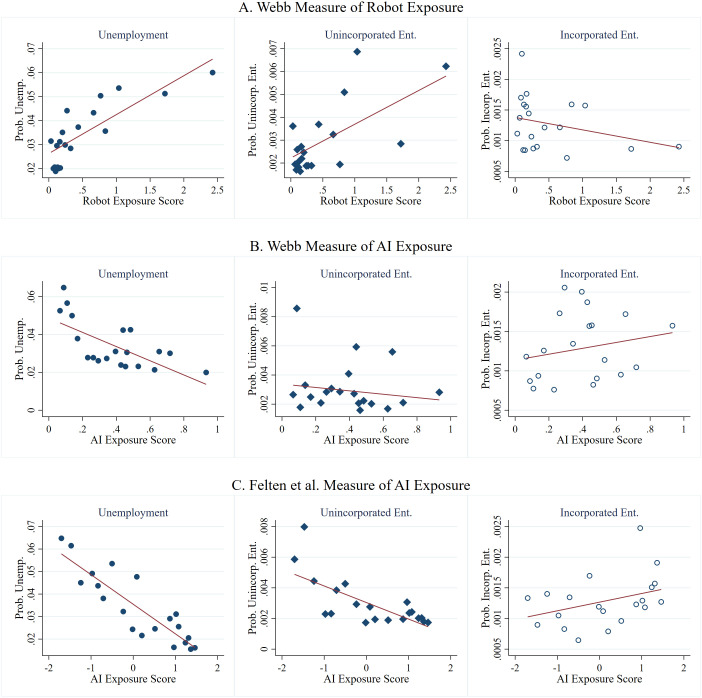
Unconditional relationship between automation technology exposure measures and labor mobility. This figure displays binned scatterplots summarizing the relationships between exposure to automation technologies and labor mobility, which include transitions to unemployment, necessity entrepreneurship, and opportunity entrepreneurship. Panels A and B use robot and AI exposure measures from Webb [11]. The third graph employs the AI exposure measure from Felten et al. [12].

In Section 4.2, we examine the relationship between automation technologies, namely, industrial robots and AI, and the transition of workers into unemployment and entrepreneurship. First, our investigation of the unconditional relationship revealed distinct mobility patterns associated with automation technologies. As depicted in Fig 4A, we found that high exposure to industrial robots is correlated with increased unemployment and a greater inclination toward necessity entrepreneurship with a lesser tendency toward opportunity entrepreneurship. Conversely, Figs 4B and 4C indicate a different scenario with AI exposure: occupations highly exposed to AI tend to have lower unemployment rates and a decreased propensity for necessity entrepreneurship with an increased likelihood of moving into opportunity entrepreneurship. This pattern persists across different AI exposure metrics, suggesting a distinct relationship between AI and job mobility compared to industrial robots.

Subsequently, we reran our main specification by displacing the API with occupation-specific exposure measures to industrial robots and AI. A potential concern with this analysis is that the industrial robot and AI exposure measures may be acting as proxies for exposure to traditional software or digital technologies in addition to exposure to industrial robots and AI. To address this concern, we included Webb’s measure [15] of exposure to “software” as a control variable. This measure was calculated in a manner similar to his AI exposure score, but it specifically targets occupations suitable for traditional software and digital technologies.

In our study, as shown in Panel A of Table 3, we observed a positive correlation between an exposure to industrial robots and a likelihood of workers’ transitioning into unemployment. Specifically, a one-standard-deviation increase in robot exposure score correlates with a 0.840 percentage point rise in the likelihood of unemployment. In relative terms, given that the baseline unemployment rate is about 3.503%, this corresponds to a 23.979% increase in the unemployment rate for employees whose jobs are highly exposed to industrial robot adoption. Similarly, we found a positive association between increased occupational exposure to industrial robots and the likelihood of transitioning into necessity entrepreneurship. Here, a one-standard-deviation increase in robot exposure corresponds to a 0.084 percentage point increase, which is 27.907% of the average monthly probability for such a transition. However, this correlation does not appear to extend to transitions into opportunity entrepreneurship.

**Table 3:** Automation technology exposure measures, unemployment, and entrepreneurship.

DV	A.Webb Measure of Robot Exposure		
	Unemployment	Necessity Ent.	Opportunity Ent.
Robot exposure	0.00840^***^	0.00084^***^	–0.00004
	(0.00194)	(0.00032)	(0.00011)
Software exposure	–0.00408	–0.00125^***^	–0.00027
	(0.00293)	(0.00039)	(0.00018)
DV	B.Webb Measure of AI Exposure		
	Unemployment	Necessity Ent.	Opportunity Ent.
AI exposure	–0.01724^***^	–0.00076	0.00009
	(0.00311)	(0.00058)	(0.00027)
Software exposure	0.01263^***^	0.00000	–0.00036^*^
	(0.00276)	(0.00041)	(0.00021)
DV	C.Felten et al. Measure of AI Exposure		
	Unemployment	Necessity Ent.	Opportunity Ent.
AI exposure	–0.00617^***^	–0.00069^***^	0.00008
	(0.00091)	(0.00015)	(0.00010)
Software exposure	0.00028	–0.00087^***^	–0.00025
	(0.00249)	(0.00030)	(0.00016)
N	1,512,965	1,512,965	1,512,965
Controls:			
Individual	Yes	Yes	Yes
State unemp. rate	Yes	Yes	Yes
Fixed effects:			
Industry	Yes	Yes	Yes
State # year	Yes	Yes	Yes
Seasonality	Yes	Yes	Yes

Sample is employees who work for private companies. Robust standard errors, adjusted for clustering at the occupation level, are presented in parentheses. Individual-level control variables are family income, citizenship, age, age squared, sex, ethnicity, marital status, education, citizenship, and household income. The automation probability index is sourced from Frey and Osborne. * p < 0.1, ** p < 0.05, *** p < 0.01

We then investigated the association between workers’ exposure to AI technologies and their transition into unemployment and entrepreneurship. As presented in Panel B of Table 3, we found employees in occupations with high AI exposure are associated with a decreased likelihood of moving into unemployment. Specifically, a one-standard-deviation increase in AI exposure is associated with a decrease of 1.724 percentage points in the probability of transitioning into unemployment. This reduction represents 49.215% of the average monthly baseline probability of transitioning into unemployment. This finding was consistent even when using an alternative AI exposure measure by Felten et al. [16], as shown in Panel C. With regard to entrepreneurship and AI exposure scores, our analysis indicated that high AI exposure in occupations correlates with a decreased likelihood of transitioning into necessity entrepreneurship. However, there is no significant correlation between AI exposure and transitioning into opportunity entrepreneurship. Thus, in sum, unlike with industrial robots, high AI exposure appears to lower the chances of unemployment and entering into unincorporated business ventures.

**Fig 5:**
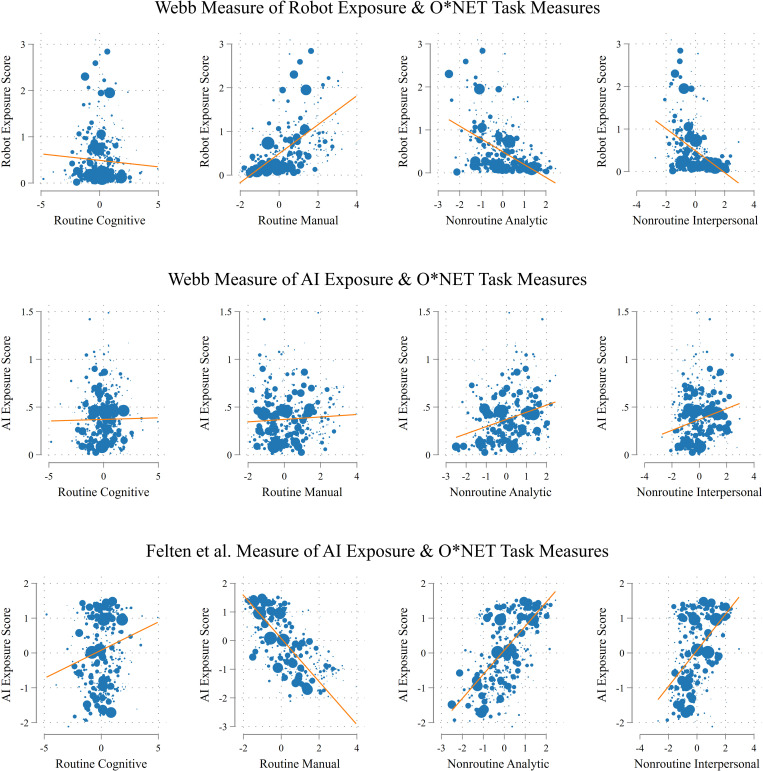
Automation technology exposure measures and O*NET task measures. *Notes:* This figure displays scatterplots summarizing the association between the automation technology exposure measures and O*NET task measures. O*NET task measures are sourced from Acemoglu and Autor [45], and automation technology exposure measures are sourced from Webb [15] and Felten et al. [16]. The size of each marker increases as the number of overlapping data points increases.

To explore the reasons why occupational exposure to different automation technologies leads to disparate labor mobility outcomes for employees, we examined the relationship between automation technologies and workers’ job tasks. As depicted in Fig 5, our analysis revealed a positive correlation between exposure to industrial robots and routine manual task scores, suggesting that jobs with higher automation exposure typically involve more repetitive manual work. Conversely, a negative correlation exists between robot exposure and nonroutine analytical and interpersonal tasks, indicating that employees with high industrial robot exposure scores are more likely to engage in repetitive manual work and routinized tasks, which seem susceptible to displacement by automation technology [2]. However, the pattern shifts when examining AI exposure. Fig 5 shows that higher AI exposure correlates with nonroutine tasks without a clear link to routine tasks. This suggests that whereas occupations with high exposure to industrial robots are more prone to job displacement due to their routine nature, those with high AI exposure involving more nonroutine tasks appear less susceptible to displacement.

### 4.3 Relationship between API and unemployment/entrepreneurship by gender

Section 4.3 investigates the association between the API and workers’ transitions into unemployment and entrepreneurship, specifically focusing on worker’s sex. To do this, we integrated a female variable as a moderator in our main analysis. The findings, detailed in Column 1 of Table 4, show that the relationship between the API and the unemployment rate does not vary significantly across genders. Additionally, the relationship between the API and opportunity entrepreneurship is not statistically significant and is similarly unaffected by the worker’s gender as shown in Column 3. However, a notable observation is that the association between the API and necessity entrepreneurship is negatively moderated by the female variable as reported in Column 2. This means that the regression line for male workers’ API in relation to necessity entrepreneurship is more pronounced than that of female workers. These results support the theoretical prediction that female workers are less likely than male workers to consider necessity entrepreneurship as an alternative career path when their jobs are at risk of automation.

**Table 4:** Automation probability index (API), unemployment, and entrepreneurship by sex.

DV	Unemployment	Necessity Ent.	Opportunity Ent.
	(1)	(2)	(3)
API	0.01146^***^	0.00141^***^	–0.00022
	(0.00238)	(0.00050)	(0.00027)
API # Female	0.00241	–0.00130^**^	0.00023
	(0.00243)	(0.00057)	(0.00024)
N	1,409,990	1,409,990	1,409,990
Adj. R^2^	0.02346	0.00356	0.00083
Controls:			
Individual	Yes	Yes	Yes
State unemp. rate	Yes	Yes	Yes
Fixed effects:			
Industry	Yes	Yes	Yes
State # year	Yes	Yes	Yes
Seasonality	Yes	Yes	Yes

Sample is employees who work for private companies. Robust standard errors, adjusted for clustering at the occupation level, are presented in parentheses. Individual-level control variables are family income, citizenship, age, age squared, sex, ethnicity, marital status, education, citizenship, and household income. The automation probability index is sourced from Frey and Osborne. * p < 0.1, ** p < 0.05, *** p < 0.01

### 4.4 Relationship between API and unemployment/entrepreneurship by COVID-19

We further explored how the association between the API and workers’ transition into unemployment and entrepreneurship may have changed following the COVID-19 outbreak. Specifically, we focused on analyzing how the relationship shifted since March 2020, which marks the onset of the COVID-19 pandemic. To consider pandemic-related factors, we controlled for workers’ exposure to COVID-19 and their physical proximity to coworkers.

The results in Table 5 examine the correlation between the API and transitions into unemployment and entrepreneurship after COVID-19 began. Column 1 indicates that from March 2020 to December 2021, workers in occupations susceptible to automation had a higher tendency to become unemployed. This result provides evidence that during the initial period of the pandemic, workers prone to automation were more likely to move to unemployment. However, after January 2022, there was no significant change in the correlation between the API and unemployment rates. This suggests that the pattern of workers in automation-prone occupations transitioning to unemployment post-January 2022 resembled the trend observed from 2015 to 2019.

**Table 5:** Automation probability index (API), unemployment, and entrepreneurship (by COVID-19).

DV	Unemployment	Necessity Ent.	Opportunity Ent.
	(1)	(2)	(3)
API	0.01110^***^	0.00057^*^	–0.00007
	(0.00204)	(0.00034)	(0.00020)
API * (2020 Mar. ~ 2021 Dec.)	0.01020^***^	0.00074^**^	–0.00025
	(0.00330)	(0.00033)	(0.00026)
API * (2022 Jan. ~ 2022 Dec.)	0.00055	0.00054	0.00014
	(0.00164)	(0.00050)	(0.00035)
API * (2023 Jan. ~ 2023 Aug.)	–0.00179	0.00088	–0.00025
	(0.00268)	(0.00073)	(0.00055)
Exposure to infection/disease index	0.00038	–0.00004	–0.00002
	(0.00127)	(0.00025)	(0.00007)
Physical proximity to coworkers index	0.00323^***^	0.00038	0.00000
	(0.00116)	(0.00030)	(0.00010)
N	1,361,252	1,361,252	1,361,252
Adj. R^2^	0.02511	0.00362	0.00084
Controls:			
Individual	Yes	Yes	Yes
State unemp. rate	Yes	Yes	Yes
Fixed effects:			
Industry	Yes	Yes	Yes
State # year	Yes	Yes	Yes
Seasonality	Yes	Yes	Yes

Sample is employees who work for private companies. Robust standard errors, adjusted for clustering at the occupation level, are presented in parentheses. Individual-level control variables are family income, citizenship, age, age squared, sex, ethnicity, marital status, education, citizenship, and household income. The automation probability index is sourced from Frey and Osborne. * p < 0.1, ** p < 0.05, *** p < 0.01

We then examined how the association between the API and workers’ transition to entrepreneurship changed. Our analysis, detailed in Column 2, reveals that from March 2020 to December 2021, workers at risk of automation displacement were more inclined to pursue unincorporated self-employment compared to the period from January 2015 to February 2020. However, this trend did not continue after 2022.

We suspect that the absence of a consistent increase in automation-prone workers’ transitioning to unemployment and necessity entrepreneurship post-2022 is likely linked to the labor market’s recovery. The initial outbreak of COVID-19 led to a sharp rise in unemployment rates, significantly constraining opportunities in the labor market and possibly driving automation-prone workers to consider necessity entrepreneurship more frequently than before as an alternative career path. However, with the widespread adoption of vaccinations, the U.S. unemployment rate gradually returned to its pre-pandemic state. Consequently, the propensity of automation-prone workers to opt for necessity entrepreneurship might have also reverted to pre-pandemic patterns. Therefore, we infer that the recovery of the labor market provided workers vulnerable to automation with opportunities similar to those available before the pandemic.

## 5 Conclusion

This study contributes to multiple research streams. First, we contribute to the literature on entrepreneurship by illuminating entrepreneurial activities of employees that are induced by firms’ operational transformations. Although previous studies have focused on the negative effects of automation technology adoption on employment [2,4,20,50], relatively little discussion has been conducted about whether employees vulnerable to automation may consider self-employment as a potential alternative in their subsequent career paths. We address this gap by empirically showing that self-employment can serve as an alternative for workers pushed out of the traditional labor market due to automation, with this transition closely linked to the nature of previously performed tasks. In particular, we show that jobs involving repetitive manual tasks are more likely to be automated, increasing the chance that affected workers turn to self-employment. This task-level perspective moves beyond the question of which occupations are being substituted by automation, highlighting instead how the specific tasks workers perform are key to understanding how automation shapes labor market outcomes. By identifying this task-based channel, our study offers a more nuanced understanding of how technological change shapes the nature and form of entrepreneurial responses.

### 5.1 Theoretical implications

Our study has several contributions. First, this study expands the literature on technological disruption and entrepreneurship [8] by exploring how workers respond to automation risks through entrepreneurial transitions. While previous research has examined the relationship between digitalization and entrepreneurship, we contribute by highlighting the heterogeneity of entrepreneurial responses to automation across different contexts. Specifically, we investigate how distinct technologies—such as AI and industrial robots—are associated with entrepreneurship, and how these effects are further shaped by demographic factors, such as gender, and by contextual changes, including the COVID-19 pandemic. By capturing these variations, our study contributes to a more nuanced and dynamic theoretical understanding of career choices amid technological disruption.

In addition, our paper extends the literature on technological adoption and employment transition [7] by showing that automation not only displaces workers but also reshapes the composition of entrepreneurship itself, increasing the share of necessity-driven, unincorporated ventures. Our findings highlight that entrepreneurship in the automation era is not always an opportunity-seeking choice but often a constrained response to structural labor market pressures. This enriches the theoretical conversation about automation’s broader socio-economic consequences, suggesting that technological progress may inadvertently drive the expansion of precarious necessity entrepreneurship, with implications for inequality and social stability.

### 5.2 Practical implications

For employers, policymakers, and educators, our findings underscore the need for targeted interventions that acknowledge the qualitative differences in entrepreneurial pathways [51]. Organizations adopting automation technologies should anticipate not only productivity gains but also the unintended consequence of pushing displaced workers into precarious self-employment. Employers can mitigate this risk by offering reskilling programs, internal redeployment opportunities, and proactive career transition support.

Entrepreneurship support programs should distinguish between necessity-driven and opportunity-driven entrepreneurs. Necessity entrepreneurs—often operating unincorporated businesses with limited growth potential—require different forms of support, including microfinance, mentoring, and social protection measures, rather than standard innovation-focused policies aimed at high-growth startups. Educational institutions and workforce development programs must also reconsider how they prepare individuals for technological disruption. Emphasizing adaptive skills, financial literacy, and informal business management training may better serve those likely to enter necessity entrepreneurship due to automation-driven displacement.

### 5.3 Social implications

From a societal perspective, the rise of automation-induced necessity entrepreneurship raises critical concerns about inequality and labor market precarity. While entrepreneurship is often celebrated as a driver of innovation and growth, our findings highlight a darker side: for many workers, especially those with lower educational attainment and fewer resources, self-employment is not an empowering choice but a last resort to avoid unemployment.

This trend risks exacerbating social and economic disparities, as individuals pushed into necessity entrepreneurship often lack access to social safety nets, health insurance, and stable income. Moreover, these precarious ventures typically have higher failure rates and limited potential for wealth accumulation, reinforcing cycles of vulnerability. Policy responses must therefore go beyond promoting entrepreneurship in general. Instead, they should focus on protecting workers from the structural consequences of technological displacement by ensuring access to social insurance, providing pathways to stable employment, and supporting inclusive forms of entrepreneurship that prioritize long-term sustainability and security.

### 5.4 Limitations and future research

Our study acknowledges several limitations and provides opportunities for future research. First, the research did not consider specific industries or occupations affected by automation. A sector-specific analysis could offer a more comprehensive understanding of the entrepreneurial dynamics driven by automation adoption. Second, although our empirical analysis found a positive relationship between occupational automation risks and the transition to entrepreneurship, it was challenging to discern the consequences of such mobility. Long-term studies may uncover the full effect of the automation adoption on entrepreneurial activities. Finally, our paper illuminated the adverse effect of technology adoption on specific workers with an emphasis on policy design to mitigate economic inequality for automation-prone workers. Future study may focus on the potential benefits of automation technologies for a balanced understanding and thoughtful approach to technological innovation.

## Supporting information

S1 Table Current population survey rotation pattern. (DOCX)

S2 Table Main independent variable description. (DOCX)

S3 Table Acemoglu and autor (2011)’s O*NET task measures. (DOCX)

## References

[1] Brynjolfsson E, McAfee A. The second machine age: Work, progress, and prosperity in a time of brilliant technologies. WW Norton & Company. 2014.

[2] Acemoglu D, Restrepo P. Robots and Jobs: Evidence from US Labor Markets. Journal of Political Economy. 2020;128(6):2188–244. doi: 10.1086/705716

[3] Brynjolfsson E, McAfee A. Winning the race with ever-smarter machines. MIT Sloan Management Review. 2011.

[4] Frey CB, Osborne MA. The future of employment: How susceptible are jobs to computerisation?. Technological Forecasting and Social Change. 2017;114:254–80. doi: 10.1016/j.techfore.2016.08.019

[5] Mokyr J, Vickers C, Ziebarth NL. The History of Technological Anxiety and the Future of Economic Growth: Is This Time Different?. Journal of Economic Perspectives. 2015;29(3):31–50. doi: 10.1257/jep.29.3.31

[6] Chernoff A, Warman C. COVID-19 and implications for automation. Appl Econ. 2023;55(17):1939–57.

[7] Filippi E, Bannò M, Trento S. Automation technologies and their impact on employment: A review, synthesis and future research agenda. Technological Forecasting and Social Change. 2023;191:122448. doi: 10.1016/j.techfore.2023.122448

[8] Fossen FM, Sorgner A. Digitalization of work and entry into entrepreneurship. J Bus Res. 2021;125:548–63.

[9] Fossen FM. Self-employment over the business cycle in the USA: a decomposition. Small Bus Econ (Dordr). 2021;57(4):1837–55. doi: 10.1007/s11187-020-00375-3 38624355 PMC7387264

[10] Agarwal R, Ganco M, Raffiee J. Immigrant entrepreneurship: The effect of early career immigration constraints on new venture formation. Organ Sci. 2022;33(4):1372–95.

[11] Dencker JC, Bacq S, Gruber M, Haas M. Reconceptualizing necessity entrepreneurship: A contextualized framework of entrepreneurial processes under the condition of basic needs. Academy of Management Review. 2021;46(1):60–79.

[12] Fairlie R, Fossen F. Opportunity versus necessity entrepreneurship: Two components of business creation. Santa Cruz (CA): University of California, Santa Cruz. 2018. https://escholarship.org/uc/item/8q51t8z1

[13] Agarwal R, Ganco M, Raffiee J. Immigrant entrepreneurship: The effect of early career immigration constraints on new venture formation. Organ Sci. 2022;33(4):1372–95.

[14] Bögenhold D. Self-employment and entrepreneurship: productive, unproductive or destructive?. Against entrepreneurship: a critical examination. London: Routledge. 2020. 19–35.

[15] Webb M. The impact of artificial intelligence on the labor market. In: 2019. https://papers.ssrn.com/sol3/papers.cfm?abstract_id=3482150

[16] Felten E, Raj M, Seamans R. Occupational, industry, and geographic exposure to artificial intelligence: A novel dataset and its potential uses. Strategic Management Journal. 2021;42(12):2195–217.

[17] Dauth W, Findeisen S, Südekum J, Woessner N. German robots-the impact of industrial robots on workers. 2017. https://papers.ssrn.com/sol3/papers.cfm?abstract_id=3039031

[18] Graetz G, Michaels G. Robots at work. Rev Econ Stat. 2018;100(5):753–68.

[19] Autor DH. Why are there still so many jobs? The history and future of workplace automation. J Econ Perspect. 2015;29(3):3–30.

[20] Arntz M, Gregory T, Zierahn U. The risk of automation for jobs in OECD countries: A comparative analysis. 2016. https://www.oecd-ilibrary.org/social-issues-migration-health/the-risk-of-automation-for-jobs-in-oecd-countries_5jlz9h56dvq7-en

[21] Nedelkoska L, Quintini G. 2018. https://www.oecd-ilibrary.org/content/paper/2e2f4eea-en

[22] Sørensen JB, Sharkey AJ. Entrepreneurship as a mobility process. Am Sociol Rev. 2014;79(2):328–49.

[23] Evans DS, Jovanovic B. An Estimated Model of Entrepreneurial Choice under Liquidity Constraints. Journal of Political Economy. 1989;97(4):808–27. doi: 10.1086/261629

[24] Arum R, Müller W. Self-Employment Dynamics in Advanced Economies. The Reemergence of Self-Employment. Princeton University Press. 2009. 1–35.

[25] Sørensen JB, Fassiotto MA. Organizations as fonts of entrepreneurship. Organ Sci. 2011;22(5):1322–31.

[26] Hwang KJ. Entrepreneurship as a bridge to employment? Evidence from formerly incarcerated individuals. Acad Manag Proc. 2021;2021(1):15966.

[27] Fairlie RW. Entrepreneurship, Economic Conditions, and the Great Recession. Economics Manag Strategy. 2013;22(2):207–31. doi: 10.1111/jems.12017

[28] Chui M, Manyika J, Miremadi M. Four fundamentals of workplace automation. McKinsey Q. 2015;29(3):1–9.

[29] Bessen J, Goos M, Salomons A, van den Berge W. Automation: A guide for policymakers. 2020. https://www.brookings.edu/wp-content/uploads/2020/01/Bessen-et-al_Full-report.pdf

[30] Fujita S. Labor Market Recovery During the COVID-19 Pandemic. Econ Insights. 2022;7(2):2–10.

[31] de Vries GJ, Gentile E, Miroudot S, Wacker KM. The rise of robots and the fall of routine jobs. Labour Economics. 2020;66:101885. doi: 10.1016/j.labeco.2020.101885

[32] Agrawal A, Gans JS, Goldfarb A. Do we want less automation?. Science. 2023;381(6654):155–8. doi: 10.1126/science.adh9429 37440634

[33] Agrawal A, Gans JS, Goldfarb A. Exploring the impact of artificial Intelligence: Prediction versus judgment. Information Economics and Policy. 2019;47:1–6. doi: 10.1016/j.infoecopol.2019.05.001

[34] Davenport TH, Ronanki R. Artificial intelligence for the real world. Harvard Business Review. 2018;96(1):108–16.

[35] Heaven WD. Google DeepMind wants to define what counts as artificial general intelligence. MIT Technology Review. 2023.

[36] Caliendo M, Fossen FM, Kritikos AS. Risk attitudes of nascent entrepreneurs–new evidence from an experimentally validated survey. Small Bus Econ. 2009;32(2):153–67. doi: 10.1007/s11187-007-9078-6

[37] Goolsbee A, Syverson C. Fear, lockdown, and diversion: Comparing drivers of pandemic economic decline 2020. J Public Econ. 2021;193:104311. doi: 10.1016/j.jpubeco.2020.104311 33262548 PMC7687454

[38] Lund S, Cheng WL, Dua A, De Smet A, Robinson O, Sanghvi S. McKinsey Global Institute. 2020;23.

[39] Petropoulos G. Automation, COVID-19, and labor markets. 2021. https://www.econstor.eu/handle/10419/238586

[40] Loewen P, Lee-Whiting B. Automation, AI and COVID-19. In: Public Policy Forum: Ottawa, Ontario. 2021. https://ppforum.ca/wp-content/uploads/2021/06/2Automation-AI-and-COVID-19-PPF-June2021-EN.pdf

[41] Georgieff A, Milanez A. What happened to jobs at high risk of automation? 2021. https://www.oecd-ilibrary.org/content/paper/10bc97f4-en

[42] Albanesi S, Kim J. Effects of the COVID-19 recession on the US labor market: Occupation, family, and gender. J Econ Perspect. 2021;35(3):3–24.

[43] Guzman J, Stern S. The state of American entrepreneurship: New estimates of the quantity and quality of entrepreneurship for 32 US states, 1988–2014. Am Econ J Econ Policy. 2020;12(4):212–43.

[44] Levine R, Rubinstein Y. Smart and Illicit: Who Becomes an Entrepreneur and Do They Earn More?*. The Quarterly Journal of Economics. 2017;132(2):963–1018. doi: 10.1093/qje/qjw044

[45] Acemoglu D, Autor D. Skills, tasks and technologies: Implications for employment and earnings. Handbook of labor economics. Elsevier. 2011. 1043–171.

[46] Autor DH, Levy F, Murnane RJ. The skill content of recent technological change: an empirical exploration. Q J Econ. 2003;118(4):1279–333.

[47] Hmieleski KM, Carr JC, Baron RA. Integrating Discovery and Creation Perspectives of Entrepreneurial Action: The Relative Roles of Founding CEO Human Capital, Social Capital, and Psychological Capital in Contexts of Risk Versus Uncertainty. Strategic Entrepreneurship. 2015;9(4):289–312. doi: 10.1002/sej.1208

[48] Azoulay P, Jones BF, Kim JD, Miranda J. Age and High-Growth Entrepreneurship. American Economic Review: Insights. 2020;2(1):65–82. doi: 10.1257/aeri.20180582

[49] Azoulay P, Jones BF, Kim JD, Miranda J. Immigration and Entrepreneurship in the United States. American Economic Review: Insights. 2022;4(1):71–88. doi: 10.1257/aeri.20200588

[50] Egana-delSol P, Bustelo M, Ripani L, Soler N, Viollaz M. Automation in Latin America: Are Women at Higher Risk of Losing Their Jobs?. Technological Forecasting and Social Change. 2022;175:121333. doi: 10.1016/j.techfore.2021.121333 PMC8628127 34866673

[51] Autor D. Polanyi’s paradox and the shape of employment growth. 2014. https://www.nber.org/papers/w20485

