# Supplementary material for: When automation hits jobs: Entrepreneurship as an alternative career path
Source: PLoS One. 2025 Sep 8;20(9):e0331244. doi: 10.1371/journal.pone.0331244 (PMC12416648; doi:10.1371/journal.pone.0331244)
Supplement: S1 Table — (DOCX) [file pone.0331244.s001.docx]

| **S1 Table.** *Current Population Survey Rotation Pattern* | | | | | | | | |
| --- | --- | --- | --- | --- | --- | --- | --- | --- |
|  | Month in Sample (MIS) | | | | | | | |
|  | 1 | 2 | 3 | 4 | 5 | 6 | 7 | 8 |
| ***Year (T)*** |  |  |  |  |  |  |  |  |
| January | A | z | y | x | o | n | m | l |
| February | B | A | z | y | p | o | n | m |
| March | C | B | A | z | q | p | o | n |
| April | D | C | B | A | r | q | p | o |
| May | E | D | C | B | s | r | q | p |
| June | F | E | D | C | t | s | r | q |
| July | G | F | E | D | u | t | s | r |
| August | H | G | F | E | v | u | t | s |
| September | I | H | G | F | w | v | u | t |
| October | J | I | H | G | x | w | v | u |
| November | K | J | I | H | y | x | w | v |
| December | L | K | J | I | z | y | x | w |
|  |  |  |  |  |  |  |  |  |
| ***Year (T+1)*** |  |  |  |  |  |  |  |  |
| January | M | L | K | J | A | z | y | x |
| February | N | M | L | K | B | A | z | y |
| March | O | N | M | L | C | B | A | z |
| April | P | O | N | M | D | C | B | A |
| May | Q | P | O | N | E | D | C | B |
| June | R | Q | P | O | F | E | D | C |
| July | S | R | Q | P | G | F | E | D |
| August | T | S | R | Q | H | G | F | E |
| September | U | T | S | R | I | H | G | F |
| October | V | U | T | S | J | I | H | G |
| November | W | V | U | T | K | J | I | H |
| December | X | W | V | U | L | K | J | I |
