# Supplementary material for: When automation hits jobs: Entrepreneurship as an alternative career path
Source: PLoS One. 2025 Sep 8;20(9):e0331244. doi: 10.1371/journal.pone.0331244 (PMC12416648; doi:10.1371/journal.pone.0331244)
Supplement: S2 Table — (DOCX) [file pone.0331244.s002.docx]

**S2 Table. Main Independent Variable Description**

In this section, we provide a more detailed explanation of the measurement we used to assess the extent of workers' exposure to automation risk and automation technology.

1. *Frey and Osborne (2017)’s measure on API:* API is designed to capture the risk of job replacement by estimating the probability of computerization in 702 detailed occupations over the next 10 to 20 years, based on expert opinion and features of selected occupations in the O*Net database. Together with a group of machine learning (ML) researchers, they first subjectively hand-labeled 70 occupations, assigning a value of 1 if the occupation is fully automatable, and otherwise 0. They also identify nine objective O*NET variables for bottlenecks in job automation. Then, they combined these two databases to construct a training dataset, which contains information about variation in the automation probability of the 70 occupations as a function of nine selected O*NET variables. Based on this training dataset, they employ ML algorithms to construct a prediction model, which estimates the probability of computerization in 702 occupations using the matrix of nine O*NET variables.
2. *Webb’s (2019) measure on occupational exposure to automation technologies:* The author develops a novel, objective metric to evaluate task exposure to automation technologies like industrial robots, software, and AI. This is achieved by comparing the text of patents with job descriptions. The process begins with selecting patents corresponding to a specific technology and extracting verb-noun pairs from their titles. Similarly, task descriptions from the ONET database are used to extract verb-noun pairs representative of various occupations. The extent of overlap between these pairs from patents and job tasks is then measured. This overlap indicates how much an occupation is involved in tasks that the technology can perform. To calculate an occupation's exposure to automation, a weighted average of these task-level overlaps is used, resulting in an automation technology exposure score. Essentially, this approach, as outlined in Webb (2019), aims to identify which tasks can be more exposed to automation technology by assessing the similarities between claims in patents in each automation technology and job descriptions in ONET.
3. *Felten et al. (2021) measures on occupational exposure to AI:* We use measurement by Felten et al. (2021) which is based on data from the AI Progress Measurement project, from the Electronic Frontier Foundation. The Electronic Frontier data identify a set of ten application areas in which AI has made progress since 2010, such as image recognition or language modeling. Felten et al. (2021) use Amazon MTurk to collect crowdsourced assessments of the relevance of each of these application areas to the 52 O*NET ability scales. The authors then construct the AI occupational impact for each O*NET occupation as the weighted sum of the 52 AI application-ability scores, where weights are equal to the O*NET-reported prevalence and importance of each ability in the occupation.
