# Supplementary material for: When automation hits jobs: Entrepreneurship as an alternative career path
Source: PLoS One. 2025 Sep 8;20(9):e0331244. doi: 10.1371/journal.pone.0331244 (PMC12416648; doi:10.1371/journal.pone.0331244)
Supplement: S3 Table — (DOCX) [file pone.0331244.s003.docx]

**S3 Table.** *Acemoglu and Autor (2011)’s O*NET Task Measures*

Acemoglu and Autor (2011) construct O*NET task measures by using components from O*NET Work Activities and Work Context. This table shows detailed information about the components.

| **O*NET task measures** | **O*NET Work Activities and Work Context** |
| --- | --- |
| Routine cognitive | 4.C.3.b.7 Importance of repeating the same tasks |
|  | 4.C.3.b.4 Importance of being exact or accurate |
|  | 4.C.3.b.8 Structured v. Unstructured work (reverse) |
| Routine manual | 4.C.3.d.3 Pace determined by speed of equipment |
|  | 4.A.3.a.3 Controlling machines and processes |
|  | 4.C.2.d.1.i Spend time making repetitive motions |
| Non-routine cognitive: analytic | 4.A.2.a.4 Analyzing data/information |
|  | 4.A.2.b.2 Thinking creatively |
|  | 4.A.4.a.1 Interpreting information for others |
| Non-routine cognitive: interpersonal | 4.A.4.a.4 Establishing and maintaining personal relationships |
|  | 4.A.4.b.4 Guiding, directing and motivating subordinates |
|  | 4.A.4.b.5 Coaching/developing others |
